# The Key Role of Exosomes on the Pre-metastatic Niche Formation in Tumors

**DOI:** 10.3389/fmolb.2021.703640

**Published:** 2021-09-14

**Authors:** Xuyang Yang, Yang Zhang, Yaguang Zhang, Su Zhang, Lei Qiu, Zixuan Zhuang, Mingtian Wei, Xiangbing Deng, Ziqiang Wang, Junhong Han

**Affiliations:** ^1^ State Key Laboratory of Biotherapy, Department of Gastrointestinal Surgery, West China Hospital, Sichuan University, Chengdu, China; ^2^ State Key Laboratory of Biotherapy and Cancer Center, Frontiers Science Center for Disease-related Molecular Network, West China Hospital, Sichuan University, Chengdu, China

**Keywords:** exosomes (EX), pre-metastastic niche, tumor, metastase, extracellular environment

## Abstract

Exosomes or other extracellular vesicles released from cells play an important role in cell-to-cell communication by transferring bio-information (DNA, coding/non-coding RNA, and proteins), which indicates parental cell status to recipient cells in the extracellular environment. Increasingly, evidence shows that tumor-derived exosomes mediate tumor pre-metastatic niche (PMN) remodeling to establish a supportive and receptive niche to promote tumor cell colonization and metastasis. Uptake of genetic information by target cells in the extracellular environment triggers epigenetic changes that contribute to PMN formation. Here, we provide a comprehensive overview of the current understanding of exosomes-mediated reprogramming of cells in PMN formation.

## Introduction

Twenty years ago, Hanahan, D. and Weinberg, R.A. published their seminal review, “Hallmarks of Cancer,” which had a strong impact on the study of cancer and the development of therapeutics. Their review demonstrated that in the primary tumor microenvironment, cancer obtained several biological capabilities to invade and progress (Hanahan and Weinberg, 2000; Hanahan and Weinberg, 2011). However, the precise mechanism of specific tumor metastasis to a predetermined metastatic organ is still unknown. In 2005, based on the discovery of the relationship between VEGFR1^+^ VLA-4^+^ hematopoietic progenitor clusters and organ-specific tumor spread, Lyden, D. first proposed the concept of the pre-metastatic niche (PMN) (Kaplan et al., 2005). After that, an increasing body of research demonstrated that the PMN is a supportive and permissive microenvironment with a critical role in tumor cell colonization in a distant specific organ. In 2016, Liu, Y. and Cao, X.T. systematically summarized the characteristics of the PMN, including inflammation, angiogenesis and vascular permeability, lymphangiogenesis, immunosuppression, organotrapism, reprogramming, and proposed the four PMN formation phases of priming, licensing, initiation, and progression (Liu and Cao, 2016a).

An important question regarding the PMN is how a tumor induces PMN formation in a specific organ. Of the various factors secreted by tumors to initiate PMN formation, exosomes are key components that carry bio-information (DNA, coding/non-coding RNA, and proteins) from parental cells to communicate with cells in the primary tumor and the microenvironment of distant organs (Becker et al., 2016; Peinado et al., 2017; Wortzel et al., 2019). As an emerging research field, the study on exosomes-mediated PMN formation is rapidly developing. This review aims to summarize the current knowledge about the progress of tumor-derived exosome-mediated PMN formation and its clinical application.

## The Biogenesis and General Function of Exosomes

Exosomes are extracellular vesicles with a diameter of 30–150 nm that can be secreted by most cells into the extracellular environment. Exosomes exist widely in blood, urine, saliva, and other bodily fluids. Unlike budding, the exosomes biogenesis is complex and involves endocytic pathways, including endosomal sorting complex required for transport (ESCRT) and non-ESCRT dependent pathways (Théry et al., 2002; Wei et al., 2021). The most unique structural feature of exosomes is a lipid bilayer membrane protecting various biomolecules (proteins, lipids, RNA, and DNA) from enzyme degradation (Théry et al., 2002). The most attractive feature of exosomes is that they carry cargo to target cells in a specific organ and thus influence the phenotype of the recipient cells. Therefore, exosomes are important mediators of cell-to-cell communication.

**FIGURE 1:**
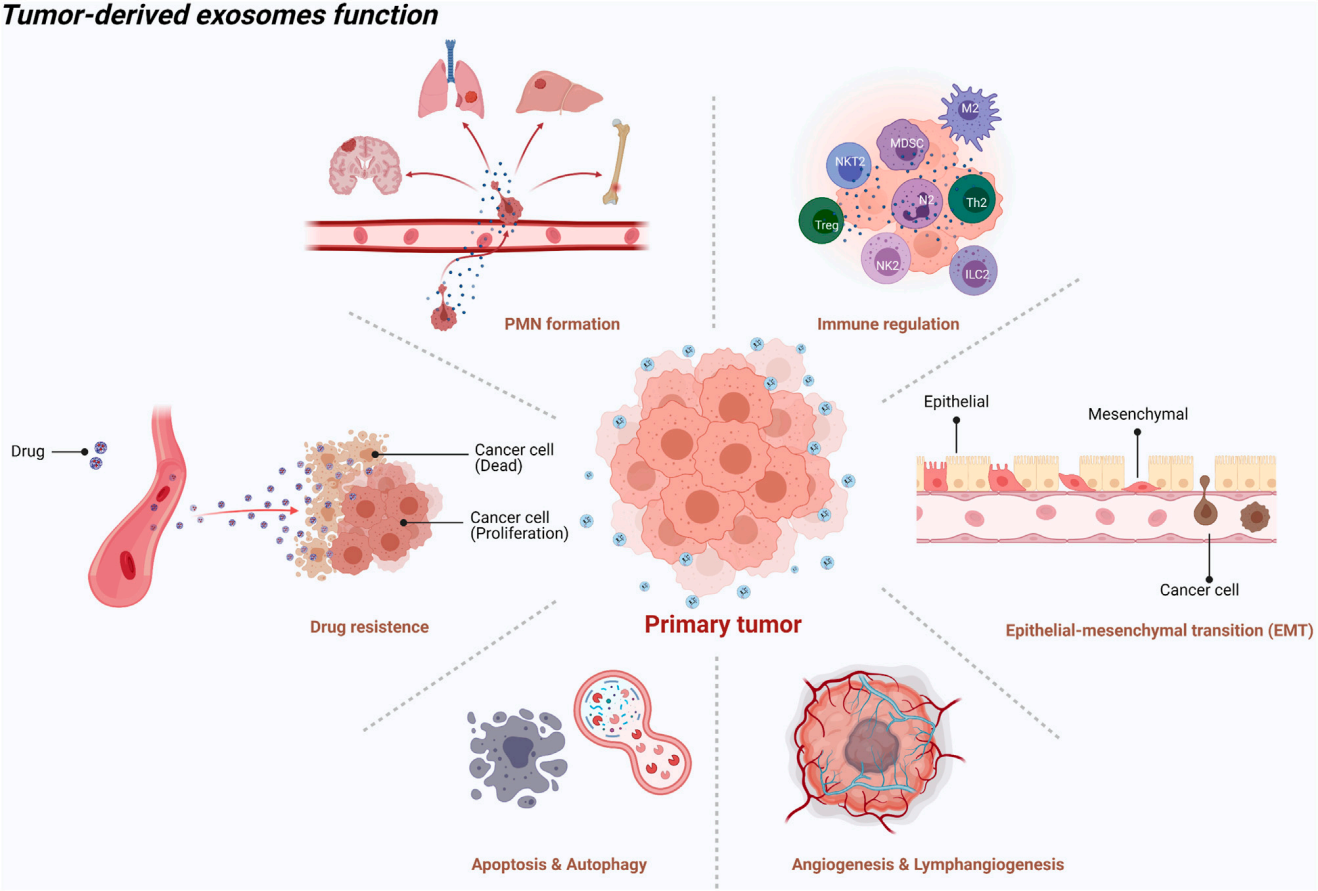
An illustration showed the tumor-derived exosomes function [created with BioRender.com (https://biorender.com/)].

**FIGURE 2:**
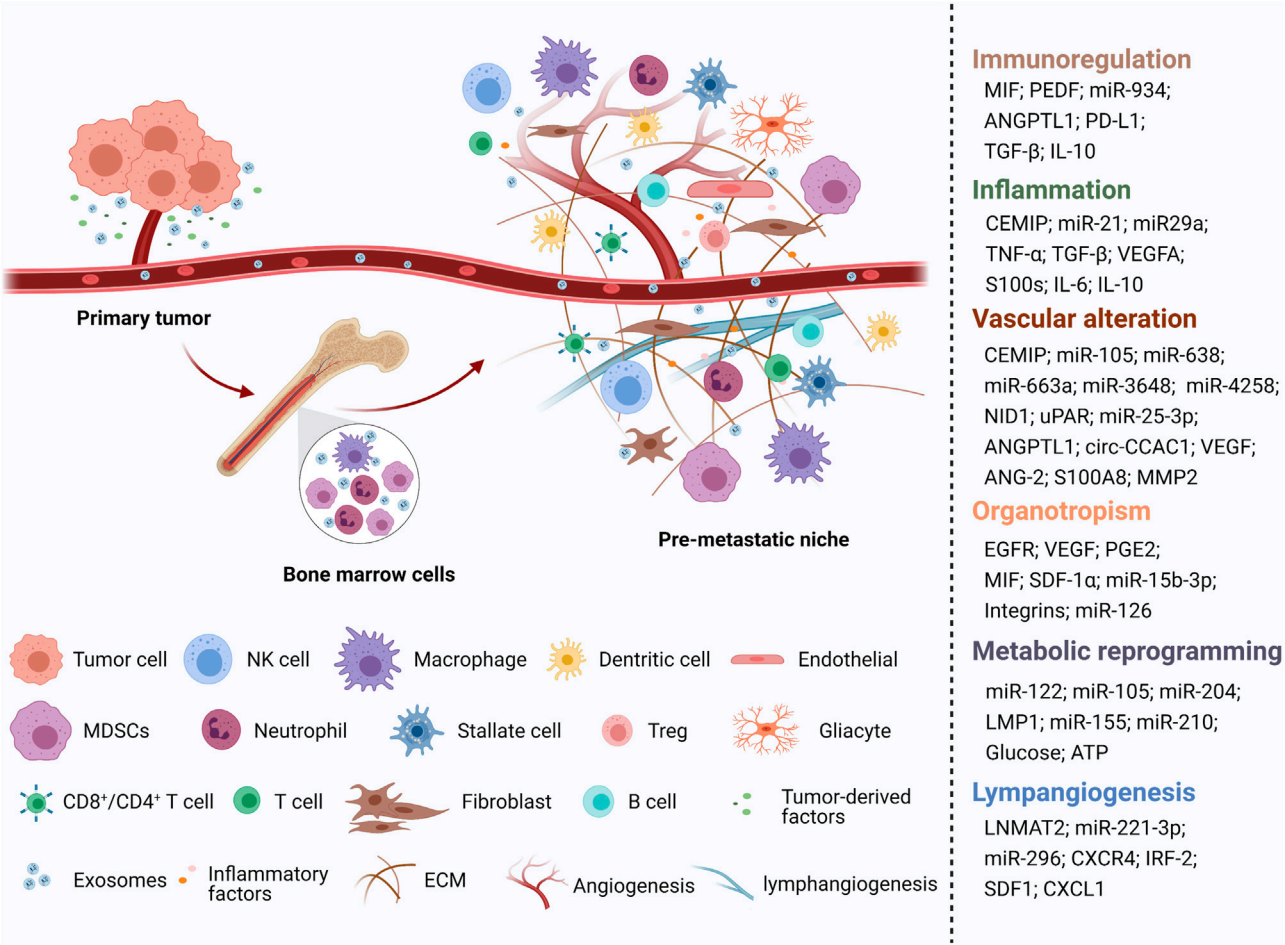
An illustration showed the role of tumor-derived exosomes in PMN formation [created with BioRender.com (https://biorender.com/)].

Generally, exosomes are reported to be widely involved in physiological and pathological processes, such as antigen presentation and immune response, cell apoptosis and senescence, material transportation and signal transduction between cells, tumor cell proliferation and invasion, and epithelial mesenchymal transition (EMT) (Théry et al., 2002) (Figure 1). Increasing evidence shows that primary tumor cells secrete exosomes that can be transferred to distant metastatic organs before the final arrival of tumor cells. This process allows for construction of a favorable, hospitable PMN that fosters the growth of disseminated tumor cells (Mo et al., 2021) (Figure 2).

## Exosomes Upregulate Inflammatory Molecules in the PMN

It’s well known that inflammation is an important driver in triggering tumor progression and metastasis according to various ways including aiding in malignant cells proliferation and survival, promoting angiogenesis, subverting adaptive immune response, and altering response to hormones and chemotherapeutic agents (Coussens and Werb, 2002; Mantovani et al., 2008). Inflammatory molecules and inflammatory cells are important components of the inflammatory microenvironment. Early in 2008, Hiratsuka, S. *et al.* found that in the pre-metastatic lung, pro-inflammatory mediators S100A8/A9 induced the expression of serum amyloid A (SAA) three and recruited Mac1^+^ myeloid cells *via* TLR-dependent manner to promote inflammatory PMN formation and facilitate metastasis (Hiratsuka et al., 2008). However, they did not reveal the role of exosomes in remodeling the inflammatory PMN formation. After that, increasing evidence reveals that tumor-derived exosomes are involved in inducing inflammatory PMN formation in distant organs to promote metastasis (Table 1). Cell migration-inducing and hyaluronan-binding protein (CEMIP) is related to hyaluronic acid depolymerization (Yoshida et al., 2013), which could promote tumor proliferation and metastasis through activating Wnt/β-catenin signaling (Birkenkamp-Demtroder et al., 2011). Breast and lung tumor-derived exosomes containing CEMIP could induce a pro-inflammatory vascular niche by upregulating cytokines Ptgs2, Tnf, and Ccl/Cxcl cytokines to promote brain metastasis. Exosomal CEMIP was proved to be able to predict brain metastasis progression and patient survival (Rodrigues et al., 2019). Tumor-secreted exosomal miR-21 and miR-29a can bind to murine TLR7 (Toll-like receptor 7) and human TLR8 (Toll-like receptor 8) in immune cells, inducing a TLR -mediated inflammatory response that may eventually provide suitable conditions for tumor metastasis (Fabbri et al., 2012). TLR7 binding to exosomal miR-21 was also reported to mediate liver macrophage polarization toward an interleukin-6 (IL-6)-secreting pro-inflammatory phenotype, thus providing a favorable environment for colorectal cancer liver metastasis (Shao et al., 2018a). Exosomes derived from metastatic melanoma cells that spontaneously metastasize to the lung and the brain could trigger proinflammatory signaling in lung fibroblasts and brain astrocytes *via* recruitment of immune cells and promote the expression of inflammation-activating factors, including Hmgb1, Tslp, and Irf1 (Gener Lahav et al., 2019). One recent study revealed that breast cancer-derived exosomal microRNA-200b-3p uptaken by alveolar epithelial type II cells (AEC II) induce the high expression of C-C motif chemokine ligand 2 (CCL2), S100A8/9, MMP9, and colony-stimulating factor 1 (CSF-1) in lung to recruit the myeloid-derived suppressor cells (MDSCs) and promote inflammatory PMN formation (Gu et al., 2021). Therefore, during the establishment of an inflammatory environment in organs to which tumors will metastasize, exosomes contribute to upregulation of pro-inflammatory cytokines and inflammation-activating factors, as well as recruitment of immune cells to the PMN.

**TABLE 1:** The role of exosomes in inflammation, lymphangiogenesis, and organotropism.

Phenotype	Type of primary cancer	Contents in exosomes	Target organ	Specific mechanism	References
Inflammation	Breast cancer/lung cancer	CEMIP	Brain	Upregulating cytokines Ptgs2, Tnf, and Ccl/Cxcl cytokines	Rodrigues et al. (2019)
	Lung cancer	miR-21, miR29a	No specific	Binding to TLR7 and human TLR8 in immune cells, inducing a TLR-mediated inflammatory response	Fabbri et al. (2012)
	CRC	miR-21	Liver	Binding to TLR7 to mediate liver macrophage polarization toward an IL-6-secreting proinflammatory phenotype	Shao et al. (2018)
	Melanoma	No specific	Lung, brain	Promoting the expression of inflammation-activating factors, including Hmgb1, Tslp, and Irf1	Gener Lahav et al. (2019)
	Breast cancer	microRNA-200b-3p	Lung	upregulating CCL2, S100A8/9, MMP9, and CSF-1 to recruit MDSCs in lung	Gu et al. (2021)
Lymphangiogenesis	OSCC	No specific	No specific	OSCC-derived exosomes stimulated the expression of VEGFs and their receptors	Morioka et al. (2016)
	Bladder cancer	LNMAT2	No specific	Stimulating HLECs tube formation and migration	Chen et al. (2020)
	CSCC	miR-221-3p	No specific	Targeting VASH1 to promote lymphangiogenesis through activating the ERK/AKT pathway	Zhou et al. (2019)
	HCC	miR‐296	No specific	Binding the HANR, promoting lymphangiogenesis via activating EAG1/VEGF-A pathway in HLECs	Shi et al. (2019)
	Melanoma	Not specified	No specific	Breaking through the undermined CD169 + macrophage layer in the subcapsular sinus	Pucci et al. (2016)
	HCC	CXCR4	No specific	Binding the SDF-1α in the HCC and upregulating the secretions of MMP-9, MMP-2	Li et al. (2018)
	CRC	IRF-2	No specific	Boosting VEGF-C secretion from macrophages to promote the formation of a lymphatic network in SLN	Sun et al. (2019)
Organotropism	Breast cancer	Integrins α6β4, α6β1, αvβ5	Liver, lung, and brain	Exosomal integrins α6β4 and α6β1 were associated with lung metastasis, while integrin αvβ5 was related to liver metastasis	Hoshino et al. (2015)
	Gastric cancer	EGFR	Liver	EGFR^+^ exosomes integrated with liver stromal cells. HGR was activated by EGFR to attract c-MET^+^ migrated cancer cells	Zhang et al. (2017)
	Pancreatic cancer	MIF	Liver	MIF^+^ exosomes target Kupffer cells and recruit BMDCs to create PMN	Costa-Silva et al. (2015)
	CCRCC	miR-15b-3p	Lung	CD103^+^ CSC exosomes specifically target lung and promote CCRCC lung metastasis, which mediated by miR-15b-3p	Wang et al. (2019)
	Breast cancer	miR-126	Lung	Integrin β4^+^ exosomes interact with surfactant protein C^+^ cancer cells; exosomal miRNA-126 suppressed lung cancer cell proliferation and migration by the inhibition of the PTEN/PI3K/AKT signaling pathway	Nie et al. (2020)

Abbreviation: CEMIP, cell migration-inducing and hyaluronan-binding protein; miRNA, microRNA; TLR7, Toll-like receptor7; TLR8, Toll-like receptor8; CRC, colorectal cancer; IL-6, interleukin-6; C-C motif chemokine ligand 2, CCL2; myeloid-derived suppressor cells, MDSCs; OSCC, oral squamous cell carcinoma; VEGF, vascular endothelial growth factor; LNMAT2, lymph node metastasis-associated transcript 2, a kind of LncRNA; HLECs, human lymphatic endothelial cells; CSCC, Cervical squamous cell carcinoma; VASH1, vasohibin-1; HCC, hepatocellular carcinoma; HANR, HCC-associated long noncoding RNA; CXCR4, CXC chemokine receptor-4; SDF-1α, stromal cell-derived factor-1α; MMPs, Matrix metalloproteinases; IRF-2, interferon regulatory factor 2; SLN, sentinel lymph node; TGF-β, transforming growth factorβ; PDAC, pancreatic ductal adenocarcinomas; CCRCC, Clear cell renal cell carcinoma; CSC, cancer stem cell; MIF, macrophage migration inhibitory factor; HGF, hepatocyte growth factor; EGFR, epidermal growth factor receptor.

## Exosomes Increase Lymphangiogenesis

In most cancers**,** lymph node metastasis is regarded as an independent prognostic factor. The 5-years overall survival (OS) in colorectal cancer patients without lymph node metastases was 87%, while the 5-years OS rate in patients with lymph node metastases decreased to 60.6% (Rosenberg et al., 2008). This is because patients with lymph node metastasis are prone to distant metastasis. Previous studies discovered that lymphangiogenesis in the PMN could promote tumor metastasis. Lymphangiogenesis was thought to initiate with the formation of lymphatic vessels, possibly triggered by VEGF-C and VEGF-D (Karnezis et al., 2012). Exosomes were also identified as lymphangiogenesis triggers functioning in a similar manner (Morioka et al., 2016) (Table 1). However, another study showed that VEGF-C was expressed at low levels in approximately 20% of bladder cancer patients with lymph node metastasis, suggesting lymphangiogenesis in these patients did not result from VEGF-C (Chen et al., 2020). Instead, the authors revealed that an exosomal long noncoding RNA (lncRNA) called lymph node metastasis-associated transcript 2 (LNMAT2) could stimulate human lymphatic endothelial cells (HLECs) tube formation and migration, and thus promote tumor lymphangiogenesis and lymph node metastasis (Chen et al., 2020). Cervical squamous cell carcinoma (CSCC) derived-exosomal miR-221-3p could target vasohibin-1 (VASH1) to promote lymphangiogenesis through activating the ERK/AKT pathway (Zhou et al., 2019). In hepatocellular carcinoma (HCC), HCC-associated long noncoding RNA (HANR) could directly bind to exosomal miR-296, resulting in suppression of lymphangiogenesis *via* inhibition of the EAG1/VEGF-A pathway in HLECs. In other words, the upregulation of HANR in HCC finally promotes the occurrence of lymphangiogenesis (Shi et al., 2019). In the progression of melanoma, melanoma-derived exosomes could break through the undermined CD169^+^macrophage layer in the subcapsular sinus and lead to cancer dissemination *via* lymphatic vessels (Pucci et al., 2016). Matrix metalloproteinases (MMPs) is well known for being able to promote tumor metastasis. A recent study indicated that hepatocarcinoma cell derived exosomal CXC chemokine receptor-4 (CXCR4) could enhance HCC migration, invasion, and lymphangiogenesis. Specifically, HLECs expressed stromal cell-derived factor-1α (SDF-1α), which bound with CXCR4 in the HCC and subsequently increased the secretion of MMP-9, MMP-2, and vascular endothelial growth factor C (VEGF-C) (Li et al., 2018). Exosomes were also proved to be linked to lymphatic network remodeling in sentinel lymph nodes (SLNs) of colorectal cancer (CRC), thus facilitating lymph node metastasis. The mechanism could involve exosomal interferon regulatory factor 2(IRF-2) from CRC boosting VEGF-C secretion from macrophages, causing promotion of the formation of a lymphatic network in SLNs (Sun et al., 2019). Overall, tumor-derived exosomes mainly target HLECs and activate ERK/AKT and/or EAG1/VEGF-A signaling pathways to promote the HLECs migration and tube formation, resulting in lymphangiogenesis. More studies on the inhibition of lymphangiogenesis to treat or prevent tumor metastasis need to be conducted.

## Exosomes Define Organotropism

Organotropism refers to certain types of cancer that tend to colonize and metastasize to specific organs under the control of a range of cellular and molecular programs (Chen et al., 2018a). For instance, liver and lung are the most common metastatic sites of CRC, while breast cancer cells mainly metastasize to the lung, bone, and brain. What is the mechanism that enables this process to happen? The earliest theory accounting for this concept is the “seed and soil” theory described by Dr. Stephen Paget in 1889. In this theory, some characterized cancer cells (the “seeds”) metastasize to certain favorable organs (the “soil”) (Paget, 1989). The most important progress came from the Lyden, D. group, which conducted exosomal proteomics and found distinct integrin expression patterns in exosomes. The exosome-derived integrins α6β4 and α6β1 were associated with lung metastasis, while integrin αvβ5 was related to liver metastasis. (Table 1). Clinical data was used to further prove that specific exosomal integrins could be used to predict organ-specific metastasis (Hoshino et al., 2015). This study expands our understanding of the mechanisms regarding organ-specific metastasis. It also highlights that communication between tumor-derived exosomal integrin and resident cells in a predicted destination was the key factor to determine organotropism (Liu and Cao, 2016b). From a clinical perspective, Chen, GY. et al. found that circulating exosomal integrin β3 from lung cancer patients with brain metastasis was associated with survival and intracranial control after whole-brain radiotherapy (Chen et al., 2021). Some studies also found other exosomal molecules that contributed to tumor-specific organ metastasis. Zhang H *et al.* revealed that gastric cancer-derived exosomal EGFR can be delivered into the liver and integrated with liver stromal cells. Then, the hepatocyte growth factor bound to the c-MET receptor on the migrated cancer cells was activated, providing a fertile microenvironment for the landing and proliferation of metastatic cancer cells. Thus, EGFR-containing exosomes promoted gastric cancer liver-specific metastasis (Zhang et al., 2017). Liver is the common metastatic organ for pancreatic cancer. Costa-Silva, B. *et al.* found that Kupffer cells uptake pancreatic ductal adenocarcinomas (PDAC) derived-exosomal macrophage migration inhibitory factor (MIF). This process induces PMN formationand promotes liver metastasis (Costa-Silva et al., 2015). Wang, L *et al.* showed that CD103^+^ CSC exosomes from clear cell renal cell carcinoma (CCRCC) can induce CCRCC specific lung metastasis (Wang et al., 2019a). It is worth mentioning that intravital imaging in a mouse model allowed for *in vivo* visualization of systemic transfer of exosomes from tumors to distant metastatic organs. This imaging provided direct evidence that tumor-derived exosomes and their cargos could mediate signal transduction between primary lesions and metastases (Zomer et al., 2015).

This organotropism characteristic piqued researchers’ interest in whether tumor-derived exosomes could serve as a carrier to transfer the drug to a specific organ. Nie, H. *et al.* determined that breast cancer-derived exosomes can be internalized by non-small cell lung cancer cells *via* the interaction between integrin β4 on exosomes and surfactant protein C on the cancer cells. Furthermore, miRNA-126 loaded breast cancer exosomes can suppress lung cancer cell proliferation and migration through the inhibition of the PTEN/PI3K/AKT signaling pathway (Nie et al., 2020). Xie, X. *et al.* tested the efficacy of using this organotropism feature to load breast cancer-derived exosomes with doxorubicin (EXO- DOX). They found that the tissue distribution and accumulation of EXO-DOX were to their companion exosomes. Importantly, the EXO-DOX inhibited breast cancer lung metastasis. This study proposed a new approach for disease chemoprevention by targeting specific organs using exosomes’ organotropism mechanism (Xie et al., 2021).

## Exosomes Induce Angiogenesis and Vascular Permeability in PMN

To facilitate distant metastasis, the tumor develops several strategies to induce angiogenesis, increase vascular permeability, or destroy vascular integrity within the PMN as an initial step for its development and subsequent metastasis. Now, it is well-established that vascular endothelial cells (ECs) are crucial for vascular remodeling. Most studies on vascular remodeling use human umbilical vein endothelial cells (HUVECs) as a laboratory model system for the study of the function and pathology of ECs. Tumor-derived exosomes mainly interact with ECs and lead to reprogramming of ECs to regulate the level of zonula occludens-1 (ZO-1), occludin, and Claudin5. These three proteins are central components of tight junctions (TJs) and comprise a major group of cell-cell adhesion complexes in ECs and epithelial cells. Both exosomes-associated non-coding RNAs and proteins are associated with vascular remodeling, especially in lung PMN (Table 2).

**TABLE 2:** The role of exosomes in angiogenesis and vascular permeability.

Phenotype	Type of primary cancer	Contents in exosomes	Target organ/cells	Specific mechanism	References
Angiogenesis	Breast cancer	miR-105	Lung/Brain microvascular ECs	Downregulating microvascular ECs expression of ZO-1; destroying the endothelial barriers and vascular integrity; increasing vascular permeability	Zhou et al. (2014)
	Breast cancer/lung cancer	CEMIP	Brain ECs/microglial cells	Upregulating cytokines Ptgs2, Tnf, and Ccl/Cxcl; promoting vascular remodeling and angiogenesis	Rodrigues et al. (2019)
	Glioblastoma	Pro-angiogenic VEGF-A factor	Brain ECs	Fostering brain ECs both angiogenesis and permeability	Treps et al. (2017)
	HCC	miR-638, miR-663a, miR-3648, miR-4258	Intrahepatic ECs	Downregulating ECs expression of ZO-1 and VE-cadherin; increasing vascular permeability	Chen et al. (2018)
	HCC	NID1	Lung fibroblasts	Enhancing angiogenesis and pulmonary endothelial permeability	Mao et al. (2020)
	Melanoma	uPAR	ECs	Enhancing VE-Cadherin, EGFR, and uPAR expression and pro-angiogenic effects	Biagioni et al. (2021)
	CRC	miR-25-3p	Liver/lung ECs	Upregulating ECs expression of VEGFR2 and downregulating the level of ZO-1, occludin, and Claudin5 by silencing KLF2/KLF4	Zeng et al. (2018)
	CRC	ANGPTL1	Liver Kupffer cells	Regulating the Kupffer cells’ secretion pattern by inhibiting the JAK2-STAT3 signaling pathway and decreasing the MMP9 expression to prevent vascular leakiness	Jiang et al. (2021)
	Cholangiocarcinoma	circ-CCAC1	HUVECs	Entering HUVECs and sequestering EZH2 to modulate SH3GL2/ZO-1/Occludin signaling and reducing the levels of TJs protein; destroying vascular endothelial barriers and inducing angiogenesis	Xu et al. (2020)
	Cervical squamous cancer	Exosomal microRNA-independent	HUVECs	Inducing endoplasmic reticulum stress in HUVECs; increasing PERK and eIF2α and downregulating TJ proteins	Lin et al. (2020)
Angiogenesis under hypoxia condition	Myeloma	miR-135b	ECs	Targeting HIF-1 and enhancing angiogenesis	Umezu et al. (2014)
	Leukemia	miR-210	HUVECs	Enhancing the tube formation in HUVECs	Tadokoro et al. (2013)
	Lung cancer	miR-23a	ECs	The accumulation of HIF-1α in ECs via directly suppressed PHD1 and 2; ZO-1 was inhibited; Vascular permeability and angiogenesis was enhanced	Hsu et al. (2017)
	Lung cancer	miR-494	ECs	Suppressing PTEN by activating Akt/eNOS pathway in ECs and reshape the angiogenesis.	Mao et al. (2015)
	ESCC	—	HUVECs	Promoting HUVECs tube formation	Mao et al. (2019)

Abbreviation: ECs, endothelial cells; ZO-1, zonula occludens-1; CEMIP, cell migration-inducing and hyaluronan-binding protein; miRNA, microRNA; VEGF, vascular endothelial growth factor; HCC, hepatocellular carcinoma; VE-cadherin, vascular endothelial-cadherin; NID1, Nidogen 1; uPAR, urokinase plasminogen activator receptor; CRC, colorectal cancer; ANGPTL1, angiopoietin-like protein 1; MMPs, Matrix metalloproteinases; circ-CCAC1,cholangiocarcinoma-associated circular RNA 1; HUVECs, human umbilical vein endothelial cells; PERK, phosphorylation of protein kinase RNA-like ER kinase; eIF2, eukaryotic translation initiation factor 2 alpha; PHD1 and 2, prolyl hydroxylase 1 and 2; ESCC, esophageal squamous cell carcinoma.

In breast cancer, exosomal miR-105 targets human microvascular endothelial cells and downregulates ZO-1. This results in destruction of the endothelial barriers, decreased vascular integrity, and increased vascular permeability during lung/brain PMN formation. Breast cancer-derived exosomal miR-105 also promotes tumor distant metastasis (Zhou et al., 2014). Recently, Rodrigues, G. *et al.* found that brain endothelial and microglial cells uptake of breast brain-tropic cell-derived CEMIP^+^ exosomes triggered the inflammation pathway (Ptgs2, Tnf, and Ccl/Cxcl upregulation) and promoted vascular remodeling and angiogenesis (Rodrigues et al., 2019). Patient-derived glioblastoma stem-like cells also can secrete exosomes carrying pro-angiogenic VEGF-A factor target human brain endothelial cells, fostering both angiogenesis and permeability in those cells (Treps et al., 2017).

Hepatocellular carcinoma (HCC) with high recurrence and metastasis is related to poor prognosis. However, the mechanism of HCC intrahepatic and extrahepatic metastasis remains unclear. Yokota, Y. *et al.* determined that HuH-7M (highly intrahepatic metastatic cell line) derived exosomal miRNAs (miR-638, miR-663a, miR-3648, and miR-4258) downregulated ECs expression of ZO-1 and vascular endothelial-cadherin (VE-cadherin) and increased vascular permeability to promote intrahepatic tumorigenesis (Yokota et al., 2021). Additionally, Mao, X. *et al.* found that metastatic HCC cell-derived exosomal nidogen 1 (NID1) activated lung fibroblasts to facilitate tumor extrahepatic metastasis *via* enhancing angiogenesis and pulmonary endothelial permeability (Mao et al., 2020). In melanoma, exosomes highly expressing urokinase plasminogen activator receptor (uPAR) were internalized by ECs, thus enhancing VE-Cadherin, EGFR, and uPAR expression and pro-angiogenic effects (Biagioni et al., 2021).

In colorectal cancer (CRC), the role of exosomes in vascular remodeling in PMN remains controversial. Zeng, Z. *et al.* found that CRC-derived exosomal miR-25-3p could be transferred to ECs, upregulating the expression of VEGFR2 and downregulating the level of ZO-1, occludin, and Claudin5 by silencing KLF2/KLF4. Thus, exosomal-miR-25-3p promotes CRC liver/lung metastasis by inducing angiogenesis and vascular leakiness/permeability in liver/lung PMN (Zeng et al., 2018). However, Jiang, K. *et al.* observed that compared with normal tissues, the level of angiopoietin-like protein 1 (ANGPTL1) was significantly downregulated in CRC tumor. After being taken up by Kupffer cells, CRC-derived exosomal ANGPTL1 regulated the secretion pattern of Kupffer cells and decreased the MMP9 expression by inhibiting the JAK2-STAT3 signaling pathway. Thus, in the liver PMN, vascular leakiness was prevented, and CRC liver metastasis was attenuated (Jiang et al., 2021).

Xu, Y. *et al.* discovered a new circular RNA-Cholangiocarcinoma-associated circular RNA 1 (circ-CCAC1), a key player in cholangiocarcinoma progression. Circ-CCAC1 was increased in cholangiocarcinoma-derived exosomes. Exosomal circ-CCAC1 entered human umbilical vein endothelial cells (HUVECs) and sequestered EZH2 to modulate SH3GL2/ZO-1/Occludin signaling and reduce TJs protein levels. Consequently, vascular endothelial barriers were destroyed and angiogenesis was induced (Xu et al., 2021). In addition, Lin, Y. *et al.* found an independent exosomal miRNA of the vascular remodeling pathway. In human cervical squamous cancer, HUVECs absorbed HeLa cell-derived exosomes and the expression levels of TJs-associated protein ZO-1 and CLDN5 were down-regulated. Meanwhile, the mRNA levels of ZO-1 and CLDN5 remained unchanged. Thus, vascular permeability increased, and tumor metastasis was promoted. Interestingly, the authors argued that exosome-induced TJ protein downregulation was independent of exosomal miRNA, the ubiquitination pathways or autophagy. Furthermore, they found thattreatment of HUVECS with exosomes induced endoplasmic reticulum stress. Treatment also resulted in dramatic elevations inphosphorylation levels of protein kinase RNA-like ER kinase (PERK) and eukaryotic translation initiation factor 2 alpha (eIF2α). This increase resulted in the downregulation of TJ protein in HUVECs (Lin et al., 2020).

It is well-known that hypoxia is a common feature of malignant tumors. Hypoxic conditions can stimulate cancer cells to produce more exosomes compared with normoxic conditions. Hypoxia-induced exosomes are essential for tumor angiogenesis in both the tumor microenvironment and PMN (Shao et al., 2018b; Kumar and Deep, 2020). For blood system diseases, exosomal miR-135b from multiple myeloma under chronic hypoxia was transferred into endothelial cells. This microRNA targeted HIF-1, thereby enhancing angiogenesis (Tadokoro et al., 2013). Hypoxic leukemia cells secreted exosomes to enhance the tube formation in HUVECs *via* miR-210 (Tadokoro et al., 2013). Hsu, Y.L. *et al.* found that exosomal miR-23a derived from lung cancer cells was significantly upregulated under hypoxic conditions, leading to the accumulation of HIF-1α in the endothelial cells by directly suppressing prolyl hydroxylase 1 and 2 (PHD1 and 2). Consequently, angiogenesis was enhanced. Additionally, the tight junction protein ZO-1 was inhibited, thus increasing vascular permeability and cancer transendothelial migration (Hsu et al., 2017). Similarly, hypoxia-induced lung cancer cells secreted exosomes carrying HIF-1-induced miR-494. When transferred into residential ECs, they reshaped angiogenesis and suppressed PTEN by activating Akt/eNOS pathway. The miR-494 antagomiR can inhibit angiogenesis and attenuate the growth of tumor xenografts in nude mice (Mao et al., 2015). Compared with normoxic cultures, exosomes from esophageal squamous cell carcinoma (ESCC) cultured under hypoxia significantly promoted HUVECs tube formation and enhanced tumor growth and lung metastasis (Mao et al., 2019).

## Exosomes Induce Immune Suppression or Immune Surveillance in the PMN

Of the all characteristics of the PMN, immunosuppression may be the most important feature. For distant colonization and metastasis, tumors need strategies to overcome immunological elimination, including establishing an immunosuppressive PMN. Immunosuppression remodeling in PMN involves a complex process and a variety of cellular components, such as T cells, natural killer (NK) cells, Treg cells, neutrophils, macrophages, and MDSCs. In this process, tumor-derived exosomes have a close relationship with those cells and serve as a double-edged sword (Table 3). Increasing evidence shows that MD contribute significantly to tumor progression in both tumor microenvironment and PMN through immunosuppression remodeling. MDSCs are a heterogeneous cell population of myeloid origin. It includes myeloid progenitor cells and immature macrophages, granulocytes, and dendritic cells. A notable characteristic of MDSCs is their presence in an activated state with the high expression of reactive oxygen, nitrogen, and arginase 1. Another important property of MDSCs is their suppression of immune cells, such as NK cells B cells and T cells. T cell inhibition is a gold standard for evaluation of MDSC function (Gabrilovich and Nagaraj, 2009; Bronte et al., 2016; Kumar et al., 2016).

**TABLE 3:** The role of exosomes in immune regulation.

Phenotype	Type of primary cancer	Contents in exosomes	Target organ/cells	Specific mechanism	References
Immune regulation	Lung cancer/Melanoma	Not mention exosomes	Lung resident fibroblasts	Lung resident fibroblasts producing fibronectin to recruit VEGFR1^+^/VLA-4^+^ HPCs to form cellular clusters and PMN	Kaplan et al. (2005)
	Pancreatic ductal adenocarcinomas	MIF	Liver kupffer cells	Kupffer cell and HSCs were activated to induced the inflammatory PMN formation and recruit macrophages and granulocytes	Costa-Silva et al. (2015)
	Lung cancer/melanoma	RNAs	lung epithelial cells	TLR3^+^ lung epithelial cells increasing chemokine secretion and recruit neutrophil to form PMN	Liu et al. (2016)
	Non metastatic melanoma	PEDF	Lung	Recruiting monocytes Ly6C^low^ and promoting them differentiation into macrophage by increasing the Nr4a1 transcription factor expression; inducing macrophage M1 polarization and recruiting NK cells to prevent lung metastasis	Plebanek et al. (2017)
	Melanoma	—	Lung	Recruiting the Ly6Clow patrolling monocytes via the BAG6/CBP/p300-p53 axis to prevent lung metastasis	Schuldner et al. (2019)
	CRC	miR-934	Liver	M2 macrophage polarization via downregulation of PTEN expression and activating the PI3K/AKT signaling pathway; activating the a CXCL13/CXCR5/NFκB/p65/miR-934 positive feedback loop	Zhao et al. (2020)
	CRC	ANGPTL1	Liver	Changing Kupffer cells secretion pattern to attenuate liver metastasis	Jiang et al. (2021)
	Head and neck cancers	-	T/NK cells	Downregulating the NKG2D expression levels in NK cells; inducing apoptosis of CD8^+^ T cells; suppressing CD4^+^ T-cell proliferation; upregulating Treg cells suppressor functions	Ludwig et al. (2017)
	Pancreatic cancer	TGF-β1	NK cells	Inducing the phosphorylation of Smad2/3 and downregulating the expression of NKG2D, CD107a, TNF-α, and INF-γ to attenuate NK cell cytotoxicity	Zhao et al. (2019)
	Melanomas	PD-L1	T cells	Suppressing CD8 T cells and the immune system function	Chen et al. (2018)
	Melanoma/prostate cancer	PD-L1	Lymph node; T cells	Suppressing T cell activity	Poggio et al. (2019)
	Wound repair	PD-L1	T cells; epidermal cells and dermal fibroblasts	Suppressing T cell activation; the migration of epidermal cells and dermal fibroblasts was promoted	Su et al. (2020)
	Lung cancer/colon cancer cells	BMDCs -derived exosomal PD-L1	T cells	Inhibiting CD8^+^ T cell proliferation and activation	Sun et al. (2020)

Abbreviation: VEGFR1, vascular endothelial growth factor receptor 1; VLA-4. integrin α4β1; PMN, pre-metastatic niche; HPCs, hematopoietic progenitor cells; MIF, macrophage migration inhibitory factor; Toll-like receptors 3, TLR3; PEDF, pigment epithelium-derived factor; CRC, colorectal cancer; ANGPTL1, angiopoietin-like protein1; TGF-β1, transforming growth factor beta1; PD-L1, programmed death-ligand 1; BMDCs, bone marrow-derived cells.

Back in 2005, David Lyden’s group first revealed that tumor-specific chemokines and/or cytokines induce fibronectin production from lung resident fibroblasts to recruit bone marrow-derived cells (BMDCs) (hematopoietic progenitor cells, HPCs). These specific BMDCs express vascular endothelial growth factor receptor 1 (VEGFR1) and VLA-4 (integrin α4β1) to form cellular clusters and a pre-metastatic permissive niche for incoming tumor cells. Their findings demonstrated the critical role of BMDCs in PMN formation; however, the role of cancer-derived exosomes was not revealed (Kaplan et al., 2005). In 2015, they further revealed that pancreatic ductal adenocarcinomas-derived exosomal macrophage migration inhibitory factor (MIF) was uptaken by mouse Kupffer cells. This changed the Kupffer cell secretory phenotype to inducing secretion of TGF-β to activate HSCs. Then, the activated HSCs promoted tumor metastatic progression by inducing inflammatory PMN formation and recruiting BMDCs (macrophages and granulocytes) (Costa-Silva et al., 2015).

The role of neutrophils recruited to the tumor microenvironment and PMN in tumor progression, angiogenesis, and invasion remains controversial (Powell and Huttenlocher, 2016). Liu, Y. *et al.* found that primary tumor (Lewis lung carcinoma or B16/F10 melanoma)-derived exosomal RNAs induce chemokine secretion and neutrophil recruitment in the lung by activating TLR3 in lung epithelial cells. Thus, lung PMN was formed and lung metastasis was promoted (Liu et al., 2016a). In contrast, Plebanek, M.P. *et al.* found that exosomes from non-aggressive, poorly metastatic melanomas block lung metastasis. Non-metastatic exosomes increase the non-classical (patrolling) monocytes Ly6C^low^ cells subpopulation, producing the anti-metastatic function. They observed that non-metastatic exosomal pigment epithelium-derived factor (PEDF) increased the Nr4a1 transcription factor expression in PMo and promoted PMo differentiation into macrophages. Additionally, non-metastatic exosomes induce macrophage M1 polarization and recruit NK cells to prevent melanoma cell metastasis (Plebanek et al., 2017). Similarly, Schuldner, M. *et al.* revealed that exosomes from B-16V cells inhibited lung metastasis by recruiting the Ly6C^low^ patrolling monocytes. Moreover, the anti-tumor effect was dependent on the BAG6/CBP/p300-p53 axis, and genetic ablation of BAG6 and disruption of this pathway can reverse the effect by recruiting tumor-promoting neutrophils to the PMN (Schuldner et al., 2019). As mentioned above, macrophages, tumor-associated macrophages, Kupffer cell M2 polarization are vital for immunosuppressive PMN remodeling. Zhao, S. *et al.* revealed that colorectal cancer-derived exosomal miR-934 induced M2 macrophage polarization by downregulating PTEN expression and activating the PI3K/AKT signaling pathway. Furthermore, M2 macrophages secreted CXCL13, activating a CXCL13/CXCR5/NFκB/p65/miR-934 positive feedback loop in CRC cells that induced immunosuppressive PMN formation and promoted CRC liver metastasis (Zhao et al., 2020). On the contrary, Jiang, K. *et al.* revealed that CRC-derived exosomal ANGPTL1 changed the Kupffer cells secretion pattern to attenuated CRC liver metastasis (Jiang et al., 2021).

Exosomes can directly suppress the function of the immune system. Earlier studies revealed that exosomes could directly inhibit NK cell function. Ludwig, S. *et al.* discovered that exosomes from head and neck cancers in patients with active disease downregulated NKG2D expression levels in NK cells and induced immune suppression (Ludwig et al., 2017). Pancreatic cancer-derived exosomal TGF-β1 induced the phosphorylation of Smad2/3 in NK cells and downregulated expression of NKG2D, CD107a, TNF-α, and INF-γ to attenuate NK cell cytotoxicity (Zhao et al., 2019). Recently, the wide clinical application of programmed death receptor 1 (PD1) and its ligand programmed death-ligand 1 (PD-L1) have attracted attention. As we know, T cell function is inhibited as PD-L1 expressed in tumor cells binds to PD1 on T cell surface. Logically, locking the PD-1/PD-L1 pathway can enhance T cell killing function and improve the immune response (Meng et al., 2015). However, the response rate of patients treated with PD-1/PD-L1 inhibitors varies greatly. Convincing evidence shows the effective role of tumor exosomes in the relatively low response rate of anti-PD-L1/PD-1 therapy (Xie et al., 2019; Daassi et al., 2020; Yin et al., 2021). Recent studies have revealed that exosomes can transfer functional PD-L1 to suppress T cell effector function and induce systemic immunosuppression. Those studies have also further revealed exosomal PD-L1 as a mechanism of tumor immune escape and immunotherapy resistance. In 2018, Chen, G. *et al.* reported their important findings on the CD8^+^ T cell and the immune system suppression properties of exosomes with PD-L1 released from metastatic melanomas. When tumor-derived exosomal PD-L1 arrived in a specific organ, immunosuppressive PMN was formed (Chen et al., 2018b). Poggio, M. *et al.* further revealed that exosomal PD-L1 suppressed T cell activity in the draining lymph node, and its genetic blockage induced systemic anti-tumor immunity and memory (Poggio et al., 2019). Recently, Su, D. *et al.* found that during wound healing, exosomal PD-L1 was specifically bound to T cells and suppressed T cell activation. This binding promoted the migration of epidermal cells and dermal fibroblastsand accelerated wound contraction upon re-epithelialization (Su et al., 2019). Notably, some researchers found that exosomes from BMDCs in tumor-bearing mice carried PD-L1, and they could inhibit CD8^+^ T cell proliferation and activation to promote tumor growth (Sun et al., 2020). In clinical application, other researchers found that CAR-T cells released exosomes carrying CAR to inhibit tumor growth. Compared with CAR-T cells, though, CAR^+^ exosomes do not express PD1. Thus, their anti-tumor effect cannot be weakened by recombinant PD-L1 treatment. These findings revealed that exosomes may be useful against tumors in future therapeutic approaches (Fu et al., 2019).

## The Role of Exosomes in Metabolism Reprogramming

One important hallmark of tumor metabolism is that tumors can induce metabolic reprogramming of residing cells, especially stromal fibroblasts in the primary tumor microenvironment, to provide nutrients to cancer cells and modulate the tumor metabolic pattern (Pavlova and Thompson, 2016). Compared with the role of exosomes in cancer cell metabolism in the tumor microenvironment, the limited evidence available so far showed that exosomes could interact with non-tumor cells or stromal cells in the PMN and induce recipient cell metabolic reprogramming (Lucchetti et al., 2020) (Table 4).

**TABLE 4:** The role of exosomes in metabolism reprogramming.

Phenotype	Type of primary cancer	Contents in exosomes	Target organ	Specific mechanism	References
Metabolism reprogramming	Breast cancer	miR-122	lung fibroblasts and brain astrocytes	Downregulating the PKM2 and GLUT1 and glucose uptake in niche cells; increasing glucose availability in cancer cells	Fong et al. (2015)
	Breast cancer	miR-105	CAFs	If nutrients are sufficient, the reprogrammed CAFs fuel adjacent cancer cells by enhancing glucose and glutamine metabolism; If not, the reprogrammed CAFs convert metabolic wastes into energy-rich metabolites	Yan et al. (2018)
	Breast cancer	miR-105, miR-204	Fibroblasts	Targeting RAGC to regulate mTORC1 signaling and alter the spectrum of *de novo* protein synthesis in fibroblasts.	Fong et al. (2021)
	Nasopharyngeal carcinoma	LMP1	CAFs	Aerobic glycolysis and autophagy in activated CAFs increasing energy-rich nutrients (lactate and β-HB) to “feed” cancer cells	Wu et al. (2020)
	Melanoma	miR-155, miR-210	Fibroblasts	Increasing aerobic glycolysis and decreasing oxidative phosphorylation in fibroblasts to favor PMN formation	Shu et al. (2018)
	Prostate cancer	CAFs-derived exosomes	Cancer cells	Under nutrient deprivation or nutrient stressed conditions, CAFs-derived exosomes inhibiting mitochondrial oxidative phosphorylation and increasing glycolysis and glutamine-dependent reductive carboxylation in cancer cells	Zhao et al. (2016)

Abbreviation: PKM2, glycolytic enzyme pyruvate kinase; CAFs, cancer-associated fibroblasts; RAGC, a component of Rag GTPases; LMP1, membrane protein 1; β-HB, lactate and β-hydroxybutyrate; PMN, pre-metastatic niche.

An important finding published in 2014 by the Wang, SE. group revealed that breast cancer-derived exosomal miR-122 was uptaken by niche cells in the PMN. This suppressed recipient cell (lung fibroblasts and brain astrocytes) glucose uptake through downregulation of the glycolytic enzyme pyruvate kinase (PKM2) and GLUT1. Thus, the nutrient competition bias leaned toward cancer cells. Glucose availability was increased in cancer cells in the PMN, and cancer proliferation and colonization were also promoted (Fong et al., 2015). The authors also showed that breast cancer-derived exosomal miR-105 induced an MYC-dependent metabolic program in cancer-associated fibroblasts (CAFs). This results in metabolic plasticity in CAFs to cope with a different condition. When nutrients are sufficient, the reprogrammed CAFs enhance glucose and glutamine metabolism to fuel adjacent cancer cells. However, when nutrients are deprived and metabolic byproducts are accumulated, the reprogrammed CAFs detoxify metabolic wastes, like lactic acid and ammonium, and convert them into energy-rich metabolites. These results demonstrate that cancer can respond to changes in the metabolic environment *via* exosomes and reprograming the stromal fibroblasts in PMN (Yan et al., 2018). Additionally, another study investigated how cancer-derived exosomes influence amino acid (AA) metabolism in fibroblasts. Breast cancer-secreted exosomes suppress AA-induced mTORC1 signaling and protein synthesis in fibroblasts. The mechanism involves exosomal miR-105 and miR-204 targeting RAGC, a component of Rag GTPases that regulate mTORC1 signaling, and altering the spectrum of *de novo* protein synthesis in fibroblasts. These results illustrate that tumor-derived exosomes could reprogram amino acid metabolism and protein synthesis in fibroblasts during periodic nutrient fluctuations (Fong et al., 2021).

Similarly, Wu, X. *et al.* found that nasopharyngeal carcinoma-derived exosomal membrane protein 1 (LMP1) can induce normal fibroblast transformation into cancer-associated fibroblasts (CAFs) *via* the NF-κB p65 pathway. Accordingly, the metabolic status of CAFs and tumor cells were also changed. In activated CAFs, aerobic glycolysis and autophagy increased. Energy-rich nutrients, including lactate and β-hydroxybutyrate (β-HB), were exported to “feed” cancer cells for the tricarboxylic acid (TCA) cycle and oxidative phosphorylation (OXPHOS). Moreover, EV packaged LMP1-activated CAFs promoted the proliferation, migration, and radiation resistance of tumor cells (Wu et al., 2020). However, the dynamic and diverse modes of metabolic programming between tumor cells and stromal resident cells are complicated. Some studies found that, the stromal cells could in turn also secrete exosomes to induce metabolic reprogramming in cancer cells. Exosomes secreted by prostate cancer patient-derived CAFs and uptake by cancer cells induced tumor cells metabolic reprogramming. In sum, mitochondrial oxidative phosphorylation was inhibited in tumor cells, whereas glycolysis and glutamine-dependent reductive carboxylation was increased. In addition, nutrient deprivation or nutrient stressed conditions resulted in tumor growth (Zhao et al., 2016). By increasing aerobic glycolysis and decreasing oxidative phosphorylation in stromal cell-dermal fibroblasts, human melanoma cell-derived exosomal miR-155 and miR-210 increased extracellular acidification to favor pre-metastatic niche formation and promotes the development of metastasis (Shu et al., 2018). These studies suggest that targeting exosomes to prevent tumor from obtaining energy resources might be an effective strategy to treat tumors.

In addition, some studies also observed that metabolites enriched in exosomes had a role in the primary tumor microenvironment remodeling including metabolism reprogramming (Yang et al., 2020). Tumor-derived exosomes transferred surface-bound proteases like glycosidases to cleave the extracellular matrix (ECM) components, resulting in ECM remodeling and facilitating tumor development (Das et al., 2019). One study showed that the purine metabolite levels in exosomes decreased in head and neck squamous cell carcinoma (HNSCC) patients with advanced cancer and nodal involvement. This report provides the first evidence that HNSCC cells shuttle purine metabolites in exosomes, with immunosuppressive adenosine being the most prominent purine (Ludwig et al., 2020). Although changes in the content and levels of purine metabolites in circulating exosomes may reflect disease progression in HNSCC patients, the detailed mechanism of how purine metabolites in exosomes affect tumor metastasis still remain unknown. Another study showed that glutamate and lactate enriched in exosomes of human mesenchymal stem cells contributed to cancer cells’ survival in hypoxic and nutrient-deficient conditions (Vallabhaneni et al., 2015). Thus, exosomal metabolites can also take an active role in the target cells, and have been shown to reprogram metabolic machinery upon uptake by cancer cells, fueling growth (Zhao et al., 2016). However, so far, there are no studies reporting metabolites enriched in exosomes to have a role in PMN remodeling yet.

## Clinical Application of Exosomes in Cancer

Liquid biopsies are emerging as a noninvasive approach for early cancer detection and monitoring of residual and recurrence disease (De Rubis et al., 2019; Yu et al., 2021). The circulating tumor-derived material used in the most common application of liquid biopsy includes circulating tumor DNA (ctDNA) and circulating tumor cells (CTCs), and exosomes. Due to the unique characteristics mentioned in the previous section, exosomes have potentially broader and complementary applications than ctDNA and CTCs.

### Diagnostic Value

Early in 1979, Taylor and colleagues discovered the presence of exosomes in the peripheral blood of ovarian cancer (Taylor and Doellgast, 1979). In 2008, this team reported 8 serum-derived special microRNA (miR-21, miR-141, miR-200a, miR-200c, miR-200b, miR-203, miR-205, and miR-214) signatures of exosomes parallel with the microRNA-expression profiles of ovarian cancer. They suggested that microRNA profiling could be conducted non-invasively (named liquid biopsy) and could accurately reflect the tumor’s profile (Taylor and Gercel-Taylor, 2008). Previous studies demonstrated that the proportion of miRNAs in exosomes is higher than that in their parent cells. Furthermore, miRNAs were preferentially, not randomly incorporated into exosomes. Generally, parent cells guide specific miRNAs into exosomes *via* a sorting mechanism (Zhang et al., 2015). Compared with free circulating miRNAs, miRNAs encapsulated in exosomes are less prone to degradation, making them more suitable circulating RNA biomarkers candidates. The diagnostic value of blood-derived exosomal miRNA signatures has been extensively described in the case of breast, lung, gastrointestinal, gynecologic, and other types of cancer (Sun et al., 2018; Drula et al., 2020).

Circular RNAs (circRNAs) are a novel class of endogenous non-coding RNAs first reported to exist in exosomes by Li et al. (2015). More than 1,000 circRNAs were identified in human serum exosomes by using RNA-seq analyses. The ratio of circRNA level to linear RNA (mainly refering to microRNAs and long non-coding RNAs) level in exosomes was approximately 6-fold higher than that in cells, suggesting greater incorporation of circRNAs into exosomes than of linear RNAs. Moreover, serum exosomal circRNAs were proved to be able to distinguish patients with colon cancer from healthy controls. Additionally, the storage of the serum at room temperature for up to 24 h only modestly influences the level of circRNAs due to the protection of exosomes and their circular structure or other special sequences. Such enrichment and stability of exosomal circRNAs implied the potential as biomarkers for cancer diagnosis. However, further investigation of the functions and characteristics of exosomal circRNAs is required (Wang et al., 2019b).

Long non-coding RNAs (lncRNAs) were also found to be enriched in exosomes. Exosomal LncRNAs have numerous functions and also showed the potential to be diagnostic biomarkers (Pathania and Challagundla, 2021). Exosome-derived lncRNA colorectal neoplasia differentially expressed-h (CRNDE-h) were reported to be able to distinguish CRC patients from benign colorectal disease patients and healthy controls with 70.3% sensitivity and 94.4% specificity. This result was superior to that of carcinoembryonic antigen (CEA). Unfortunately, patients with high exosomal CRNDE-h levels had lower 5-year OS rates because they were significantly more likely to develop CRC regional lymph node metastasis and distant metastasis (Liu et al., 2016b).

Some studies reported the existence of DNA in exosomes. One study indicated that double-stranded DNA (dsDNA) in exosomes could represent the whole genomic DNA. The study’s experiments on mouse models proved that exosomal dsDNA were able to detect the BRAF (V600E) mutation in the serum isolated from melanoma-bearing mice. This result suggested the possibility of exosomal dsDNA to act as biomarkers for cancer genetic mutations, which may provide individualized protocols for targeted therapy (Thakur et al., 2014).

Proteins enriched in exosomes also had clinical diagnostic value. In 2015, Melo SA *et al.* revealed that circulating exosomal glypican-1 (GPC1) was detected in the serum of patients with pancreatic cancer. Exosomal GPC1 was significantly related to tumor burden and the survival of patients because it distinguishes healthy people from pancreatic cancer patients. Interestingly, mutant KRAS mRNA was only detected in GPC1 positive circulating exosomes (Melo et al., 2015). Therefore, diagnosis of pancreatic cancer in the early stage may be possible *via* exosomal GPC1 detection. It may also serve as a non-invasive tool to monitor the patient’s condition. The correlation between GPC1 positive circulating exosomes and KARS mRNA could also provide a basis for the choice of targeted therapy drugs. Recently, many patents about diagnostic methods based on exosomal markers have emerged. The biomarkers used in these patents are predominantly non-coding RNAs (mainly referring to microRNAs). Collectively, non-coding RNAs (miRNAs, circRNAs, lncRNAs), double-stranded DNAs, and proteins were all discovered to be present in exosomes, revealing their potential to be diagnostic biomarkers.

### Prognosis Prediction

Besides diagnostic values, exosomes are also reportedly predictors of cancer patient prognoses. A study indicated that the amount of exosomes derived from serum of CRC patients was significantly higher than in healthy controls. Moreover, high-level serum exosomes correlated with high levels of CEA and more poorly differentiated tumors. Although there was no significant difference, patients with high levels of serum exosomes tended to have shorter OS than patients with low levels of serum exosomes (3-years OS 55% vs 89%) (Silva et al., 2012). Another study further conducted plasma exosomes quantification from NSCLC patients, revealing that exosome level was significantly associated with tumor stage and 3-years OS (Liu et al., 2018). In addition, a different research team reported that low levels of circulating exosomal miR-548c-5p in CRC patients correlated with liver metastasis and later TNM stages. Not surprisingly, circulating exosomal miR-548c-5p was also significantly related to shorter 5-year OS, despite adjustment of common confounders such as age, sex, tumor differentiation status, vascular infiltration, tumor stage, lymph node metastasis, and liver metastasis (Peng et al., 2018). In patients with gastric cancer, plasma exosomal miR-23b was found to be significantly lower than healthy controls. MiR-23b demonstrated a significant association with tumor size, depth of invasion, liver metastasis, and TNM stage. Cox multivariate analysis revealed that patients with lower exosomal miR-23b had a significant shorter 5-year OS and disease-free survival (DFS) at each tumor stage (Kumata et al., 2018). Through quantitative real-time polymerase chain reaction (qPCR) and immunohistochemistry (IHC), exosome-derived B-cell translocation gene 1 (BTG-1) enrichment was located in the para-cancerous tissues of NSCLC. Furthermore, plasma exosome-derived BTG-1 levels were negatively related to tumor diameter, stage, metastasis, the degree of tumor differentiation, and abnormal CEA levels, thus leading to a poor 3-year DFS and OS (Wan et al., 2021). Collectively, the level of exosomes or exosomal non-coding RNAs and proteins is closely related to the prognosis of cancer patients. While many well-known factors like age, sex, clinicopathological stage of cancer, lymph node metastasis and distant organ metastasis affect cancer patient prognoses, identifying novel biomarkers with high sensitivity and specificity, could help clinicians make correct decisions.

### Therapeutic Implication

In recent years, researchers have searched for novel exosomal applications to treat disease, especially tumors. Inhibiting exosome release has been proved to suppress tumor proliferation, metastasis, and chemoresistance mediated by increased exosomes in cancer. For example, inhibition of CAFs-derived exosomes secretion decreased the percentage of CSCs and suppressed tumor proliferation. It was also associated with enhancing the chemotherapy sensitivity (Hu et al., 2015). Intriguingly, mast cell stabilizer ketotifen could inhibit cancer cells secreting exosomes and enhance the sensitivity of cancer cells to doxorubicin (Khan et al., 2018).

Inhibiting the process of exosome uptake is another way to limit upregulated proliferation and exosome-mediated metastasis. Recent work indicated that the anti-hypertensive drug reserpine could inhibit the uptake of tumor-derived exosomes and influence the formation of PMN, thus suppressing metastasis (Ortiz et al., 2019). Another study reported that exosomes released from EBV-infected B cells could be internalized by recipient cells and mediate phenotypic alteration. The authors further established that exosome internalization was conducted *via* a caveola-dependent endocytosis pathway, meaning that knockdown of caveolin-1 can significantly suppress the internalization of exosomes. This protein may be a promising therapeutic target in cancer therapy (Nanbo et al., 2013). Likewise, GTPase inhibitor dynasore may serve as a potential drug for chemotherapy-resistant lymphoma patients because it blocks the target cell from internalizing exosomes (Hazan-Halevy et al., 2015).

Recently, researchers explored the feasibility of using exosomes as a vehicle to deliver anti-tumor drugs. Exosomes have potential as drug carriers for three main reasons. Firstly, their double membrane structure can protect drugs from degradation. Secondly, exosomes can be selectively transported to specific organs and tissues, thus may provide a novel idea for targeted therapy. Thirdly, exosomes with low toxicity, are widespread in extracellular fluid, blood, saliva, semen, breast milk, and even penetrate the blood-brain barrier (Chen et al., 2016).,Exosomes loaded with doxorubicin were experimentally delivered specifically to tumor tissues in nude mice, possibly leading to tumor growth inhibition without overt toxicity (Tian et al., 2014). Another study using the same drug indicated that breast cancer-derived exosomes loaded with doxorubicin can inhibit breast cancer metastasis to the lungs (Tian et al., 2014). Researchers have investigated exosome delivery of other cargo as well. Interestingly, paclitaxel packed in exosomes could enhance cytotoxicity more than 50 times in drug-resistant cancer cells (Kim et al., 2016). Inspired by the organotropism of breast cancer lung metastasis, a study suggested that miRNA-126 loaded exosomes derived from breast cancer cells strongly inhibited lung cancer cell proliferation and migration. This occurred *via* suppression of the PTEN/PI3K/AKT signaling pathway. Moreover, miRNA-126 loaded exosomes could inhibit the formulation of lung metastatic foci *in vivo* (Nie et al., 2020). Collectively, inhibiting either the process of exosome release by cancer cells or uptake by target cells is an efficient strategy to suppress cancer proliferation and metastasis. Gradually, the exosome has gradually revealed its capacity as a drug-loading vehicle that may make great contributions to molecular targeted therapy and precision medicine.

Overall, exosomes have revealed their potential as diagnostic or prognostic markers. They may even also play vital roles in targeted cancer therapy. However, their complex composition, uncertain biological functions, and even safety concerns limit their translation into clinical applications. Further research is needed to transform basic research results of exosomes into clinical applications.

## Conclusion

In summary, studies have increasingly confirmed that tumor-derived exosomes have multiple functions in PMN remodeling to promote distant metastasis. However, the cargo, function, and mechanism of exosomes still need to be further explored. Some important issues need to be solved urgently. For example, what is the interaction effect between tumor-derived exosomes and stromal/immune cell-derived exosomes in the PMN? Do the different components enriched in exosomes interact with each other? How long-lasting is the effect of tumor-derived exosomes on the PMN? How reliable and valid are tumor-derived exosomes as biomarkers? How feasible is the prospect of clinical application of tumor-derived exosomes? We believe there is strong evidence that these challenges will be answered.
